# Psychological Outcomes of a Cognitive Behavioral Therapy for Youth with Inflammatory Bowel Disease: Results of the HAPPY-IBD Randomized Controlled Trial at 6- and 12-Month Follow-Up

**DOI:** 10.1007/s10880-019-09649-9

**Published:** 2019-09-10

**Authors:** Luuk Stapersma, Gertrude van den Brink, Jan van der Ende, Eva M. Szigethy, Michael Groeneweg, Frederieke H. de Bruijne, Manon H. J. Hillegers, Johanna C. Escher, Elisabeth M. W. J. Utens

**Affiliations:** 1 Department of Child and Adolescent Psychiatry/Psychology, Erasmus MC-Sophia Children’s Hospital, P.O. Box 2060, 3000 CB Rotterdam, The Netherlands; 2 Department of Pediatric Gastroenterology, Erasmus MC-Sophia Children’s Hospital, Rotterdam, The Netherlands; 3 Department of Psychiatry, University of Pittsburgh, Pittsburgh, PA USA; 4 Department of Pediatrics, Maasstad Hospital, Rotterdam, The Netherlands; 5 Department of Gastroenterology, Maasstad Hospital, Rotterdam, The Netherlands; 6 Research Institute of Child Development and Education, University of Amsterdam, Amsterdam, The Netherlands; 7 Academic Center for Child Psychiatry the Bascule/Department of Child and Adolescent Psychiatry, Academic Medical Center, Amsterdam, The Netherlands

**Keywords:** Inflammatory bowel disease, Adolescents, Young adults, Anxiety, Depression, Psychological outcomes, Cognitive behavioral therapy

## Abstract

Youth with inflammatory bowel disease (IBD) often experience psychological difficulties, such as anxiety and depression. This randomized controlled study tested whether a 3-month disease-specific cognitive behavioral therapy (CBT) in addition to standard medical care versus standard medical care only was effective in improving these youth’s psychological outcomes. As this study was aimed at prevention, we included 70 youth (10–25 years) with IBD and symptoms of subclinical anxiety and/or depression, and measured psychological outcomes at 6- and 12-month follow-up. In general, participants in both groups showed improvements in anxiety, depression, health-related quality of life, social functioning, coping, and illness perceptions, sustained until 12 months follow-up. Overall, we found no differences between those receiving additional CBT and those receiving standard medical care only. We assume that this can be explained by the perceived low burden (both somatically and psychologically) or heightened awareness of psychological difficulties and IBD. *ClinicalTrials.gov* NCT02265588.

Inflammatory bowel disease (IBD) is a chronic inflammatory disorder of the gastrointestinal tract characterized by periods of active inflammation (with increased clinical symptoms) followed by periods of clinical remission. The two main types of IBD are Crohn’s disease (CD) and ulcerative colitis (UC). Symptoms are abdominal pain, bloody diarrhea, fatigue, fever, and weight loss (Griffiths, 2004; Rose, Dhawan, & Saeed, 2015). Pediatric patients may also show anorexia/loss of appetite, malnutrition, and delayed growth and puberty onset—especially those with CD (Adamiak et al., 2013; Sauer & Kugathasan, 2009).

Adolescents and young adults (hereafter referred to as youth) with IBD may experience various psychological problems related to the disease and its treatment. Firstly, they are at risk for anxiety and depression (Brooks et al., 2016; Greenley et al., 2010). More specifically, a large cohort study found that these youth have a higher risk for anxiety or depressive disorders (Loftus Jr et al., 2011). Secondly, they are at risk for a lower health-related quality of life (HRQOL) compared to that of healthy peers (Ross, Strachan, Russell, & Wilson, 2011), likely on account of more maladaptive avoidant coping (Van der Zaag-Loonen, Grootenhuis, Last, & Derkx, 2004; McCombie, Mulder, & Gearry, 2013). In addition, negative illness perceptions (i.e., negative cognitions on for example the consequences of the disease or personal control) are associated with more negative outcomes in these youth (Knowles, Wilson, Connell, & Kamm, 2011; Rochelle & Fidler, 2013). Thirdly, youth with IBD also experience sleep problems (Manhart, Hellmann, Hamelmann, & Schlarb, 2016), related to anxiety, and depression (Pirinen, Kolho, Ashorn, & Aronen, 2014). Lastly, a study found that the social functioning of youth with IBD was lower than that of healthy controls (Greenley et al., 2010). In conclusion, youth with IBD are likely to experience psychological problems as described above, and the interrelationships between these problems make both somatical and psychological treatment of these youth even more complex.

Importantly, psychological problems of youth with IBD can influence their medical outcomes, creating a vicious circle of problems (e.g., Mikocka-Walus, Pittet, Rossel, von Kanel, & Swiss IBD Cohort Study Group, 2016a; Sweeney et al., 2018; Van Tilburg et al., 2017). A reciprocal relationship between these psychological problems and clinical symptoms of gut inflammation seems to exist (Gracie, Guthrie, Hamlin, & Ford, 2018). It has been hypothesized that psychological interventions may positively influence the inflammatory disease course (Bonaz and Bernstein, 2013). Psychological treatment should be focused on decreasing anxiety and depression and addressing other psychological problems, such as coping or negative illness perceptions, and on improving HRQOL and daily functioning. A recent randomized controlled trial (RCT) of Levy et al. (2016) in pediatric patients with IBD tested the effect of a three-session social learning and cognitive behavioral therapy (SLCBT) versus that of educational support (ES; focusing on the gastrointestinal system, food labels, and nutrition) on a large set of psychological outcomes. SLCBT outperformed educational support in improving IBD-related quality of life 1 week after treatment and coping and school attendance over the course of 12 months, but had no beneficial effect on anxiety, depression, and functional disability. However, the study participants had not been selected on either somatic of psychological symptoms, and therefore, many of them did not have psychological problems. Mikocka-Walus, Andrews, & Bampton (2016b) have suggested that targeted psychological treatment may be more useful to tackle psychological problems such as elevated anxiety and/or depression. Furthermore, Levy et al. (2016) used only three sessions, whereas in IBD it has been shown that a full 12-session protocol of disease-specific CBT improved depressive symptoms and HRQOL (Szigethy et al., 2014; Thompson et al., 2012).

We performed a RCT named HAPPY-IBD and aimed to test the extent to which disease-specific CBT in youth with subclinical anxiety and depression is effective to decrease these psychological problems (anxiety and depression, but also the other above-mentioned psychological factors). A previous study found that CBT was effective in decreasing subclinical depression in youth with IBD (Szigethy et al., 2007; Thompson et al., 2012). The ultimate objective of providing CBT specifically for these subclinical symptoms was to improve the disease course and to prevent the development of clinical anxiety and depressive disorders. Thus, the study was aimed at secondary prevention. To cover the important life phase of transition from adolescence to adulthood, we included youth aged 10–25 years. In this life phase, IBD can affect the psychological development, such as becoming independent from parents, developing long-term friendships, and forming an own (sexual) identity. For teenagers, important changes are starting secondary education, making new friends at a new school, becoming more independent from parents, and spending more time with peers. For late adolescents, these processes continue and graduating, experimenting with alcohol or drugs, finding a (side) job and earning money, and forming an identity will happen well. Young adults face developmental challenges such as finding a job, leaving home, having long-lasting romantic relationships, and becoming financially independent (Arnett, 2010). A diagnosis of IBD can involve a sense of loss in, for example, body image, future plans, self-confidence, sense of control, and roles inside and outside the family context (Szigethy, McLafferty, & Goyal, 2011). These changes and challenges should be considered in the treatment of youth with IBD.

Earlier we have reported the results of the immediate post-treatment assessment of this RCT, 3 months after baseline: youth in both the CBT and the standard medical care group showed improvements in anxiety, depression, and HRQOL, to a similar level in each group (Stapersma et al., 2018). Considering that IBD has a fluctuating disease course, we re-assessed the psychological outcomes at 6 and 12 months. We expected that patients who had received CBT would be better able to deal with possible flares and be better equipped with skills to prevent worsening of their subclinical psychological problems. Since CBT in general aims to improve anxiety and depression, these were chosen as primary outcomes. In addition, in the current study, we extended and innovated the range of our outcomes and also measured HRQOL, social functioning, coping (Kendall et al., 2016), illness perceptions (Christensen, Frostholm, Ornbol, & Schroder, 2015), and sleep problems.

In summary, the present study aims to test the effectiveness of a full disease-specific CBT protocol in addition to standard medical care, 6 and 12 months after the baseline assessment, to improve anxiety and depressive symptoms, and other psychological outcomes, in youth with IBD (10–25 years old) and subclinical symptoms of anxiety and depression, compared to standard medical care only. We hypothesized that patients who had received CBT would have more sustained improvement on all psychological outcomes than those in the standard medical care group.

## Methods

For details of this RCT and the 3-month outcomes, see Van den Brink et al. (2016) and Stapersma et al. (2018). This study was a two-armed multi-center parallel group RCT, comparing a disease-specific CBT (Primary and Secondary Control Enhancement Training for Physical Illness; PASCET-PI; Szigethy et al., 2007) in addition to standard medical care to standard medical care only (care-as-usual, CAU). The latter represented the current usual care for youth with IBD in the Netherlands, and was therefore chosen as control condition. Patients were consecutively recruited between October 2014 and October 2016 in two academic and four community hospitals in urban and rural regions. The trial design adhered to the CONSORT guidelines for non-pharmacological treatments (Boutron et al., 2017). The research protocol was approved by the Medical Ethics Committee of the Erasmus MC (approval number NL49147.078.14) and confirmed by the ethics boards of all participating hospitals. The study was registered with ClinicalTrials.gov as study number NCT02265588.

### Participants and Assessment Procedure

After patients and/or their parents had provided written informed consent, they were included in two steps. Youth from the age of 12 years provided informed consent themselves as well; the 10- and 11-year olds provided assent. Parents provided informed consent for youth up to 18 years. All participants received as an incentive a 25 EUR voucher.

*Step 1* involved baseline screening of anxiety and depression symptoms, for which all consecutive youth (aged 10–25 years) with a confirmed diagnosis of IBD (CD, UC, or inflammatory bowel disease unclassified; IBD-U) recruited in the above-mentioned period were eligible.

*Step 2*, the actual RCT, included only youth with subclinical anxiety or depression established in step 1, as we aimed to examine whether the disease-specific CBT could prevent clinical anxiety and/or depression. For that matter, it is unethical to withhold treatment to patients with clinical anxiety and/or depression.

The presence of subclinical anxiety or depressive symptoms was defined as a score equal or above the cutoff of age-appropriate questionnaires, but not meeting criteria for clinical anxiety and depression (see below). Subclinical anxiety symptoms were measured with the Screen for Child Anxiety-Related Emotional Disorders (SCARED; 10–20 years; cutoff ≥ 26 for boys and ≥ 30 for girls; Bodden, Bögels, & Muris, 2009) and the Hospital Anxiety and Depression Scale-Anxiety Scale (HADS-A; 21–25 years; cutoff ≥ 8; De Croon, Nieuwenhuijsen, Hugenholtz, & Van Dijk, 2005). Subclinical depressive symptoms were measured with the Child Depression Inventory (CDI; 10–17 years; cutoff ≥ 13; Timbremont, Braet, & Roelofs, 2008) and the Beck Depression Inventory-second edition (BDI-II; 18–25 years; cutoff ≥ 14; Van der Does, 2002).

These youth were assumed to suffer from clinical anxiety or depression if they met DSM-5 criteria for an anxiety or depressive disorder, as assessed with a psychiatric interview (Anxiety Disorders Interview Schedule for Children; ADIS-C; Siebelink and Treffers, 2001), and if they scored equal to or above the clinical cutoff on age-specific severity rating scales: the Pediatric Anxiety Rating Scale (PARS; 10–20 years; cutoff ≥ 18; Ginsburg, Keeton, Drazdowski, & Riddle, 2011) or the Hamilton Anxiety Rating Scale (HAM-A; 21–25 years; cutoff ≥ 15; Hamilton, 1959; Matza, Morlock, Sexton, Malley, & Feltner, 2010) for anxiety; the Child Depression Rating Scale Revised (CDRS-R; 10–12 years; cutoff ≥ 40; Poznanski et al., 1984); the Adolescent Depression Rating Scale (ADRS; 13–20 years; cutoff ≥ 20; Revah-Levy, Birmaher, Gasquet, & Falissard, 2007); or the Hamilton Depression Rating Scale (HAM-D; 21–25 years; cutoff ≥ 17; Hamilton, 1960; Zimmerman, Martinez, Young, Chelminski, & Dalrymple, 2013) for depression. All above-mentioned cutoffs only served for inclusion of patients and not for analysis purposes.

Youth with clinical anxiety or depression were referred to mental health care. Youth with subclinical anxiety or depressive symptoms (but not clinical anxiety or depression) were randomized at a ratio 1:1 to receive either PASCET-PI in addition to CAU or CAU only.

### Randomization

An independent biostatistician provided a computer-generated blocked randomization list with randomly chosen block sizes (with a maximum of 6) and stratification by center using the blockrand package in the R software package, thereby providing numbered envelopes per center. Participants were enrolled by a single investigator (GB). The interviewer (LS) and treating physicians had no access to the files in which the randomization result was described. We requested the youth and their parents not to reveal the trial arm assignment to the interviewer and treating physicians. Youth and parents received a link to web-based questionnaires, to be completed at home. They completed the same set of questionnaires at baseline (no longer than 2 weeks before the start of the PASCET-PI), and at the post-assessments (3, 6, and 12 months after baseline). For both groups, assessments were performed at comparable time points (i.e., between 11 and 13 weeks, 25 and 27 weeks, and 51 and 53 weeks after randomization).

### Intervention

The PASCET-PI is a disease-specific CBT protocol for youth with IBD (Szigethy et al., 2007), consisting of ten weekly individual sessions, delivered in 3 months. It was provided in a ‘blended format’: six sessions were face-to-face with a psychologist (in the young person’s own hospital), four sessions by telephone. In addition, parents of youth ≤ 20 years were invited for three face-to-face family sessions. Booster sessions were delivered by telephone 4, 5, and 6 months after baseline. The authorized Dutch translation of the PASCET-PI was used, which has been established by the research team. Originally, the PASCET-PI is targeted at depression. For this study, the treatment content was adjusted to also target aspects of anxiety such as anxiety hierarchy, exposure, cognitive restructuring, and to also target young adults (with more age-appropriate exercises and lay-out). A more detailed description is provided in Appendix 1, Table 3 or van den Brink et al. 2016. In short, sessions are focused on discussing, in an age-attuned manner, the illness narrative and the link between behavior and feelings, relaxation, discussing negative thoughts and cognitive restructuring, and on personalizing the taught skills. The therapists provided age-appropriate information and exercises. In this way, the protocol took into account the youth’s psychological, cognitive, and social development.

The therapy was provided by licensed (healthcare/CBT) psychologists with ample experience working with youth, who had all been trained by the developer (ES) and received monthly supervision by EU (clinical psychologist/professor). Treatment integrity was ensured by supervision of the therapists and by rating of audiotaped sessions. For details, see Stapersma et al. (2018). CAU consisted of regular medical consultations of 15–30 min with the (pediatric) gastroenterologist and/or IBD nurse every 3 months, in which overall wellbeing, disease activity, and future diagnostic/treatment plans were discussed.

### Outcome Measures (Online Questionnaires)

*Demographic data* were obtained from a semi-structured questionnaire (Utens, van Rijen, Erdman, & Verhulst, 2000). Socioeconomic status was based on parents’ occupational level or, for youth living on their own, the own occupational level. We classified socioeconomic status into low, middle, and high (Statistics Netherlands, 2010). Ethnicity was based on the mother’s country of birth or, if the mother was born in the Netherlands, the father’s country of birth (Statistics Netherlands, 2000). Disease characteristics were extracted from the electronic medical charts.

*Symptoms of anxiety* were assessed with the SCARED (for 10–20 years), and the anxiety scale of the HADS (for 21–25 years). Both are self-report questionnaires. The SCARED has 69-items with 3 response categories (0–2, total score 0–138; Muris, Bodden, Hale, Birmaher, & Mayer, 2011). The anxiety scale of the HADS has 7-items with 4 response categories (0–3, total score 0–21; De Croon et al. 2005). Internal consistency at baseline and the three follow-up assessments was .86, .92, .94, and .94, respectively, for the SCARED, and .54, .77, .81, .80, respectively, for the HADS-A. Clinical anxiety was established from a psychiatric interview and severity rating scales (as described above in the assessment procedure).

*Symptoms of depression* were assessed using the CDI (for 10–17 years) and the BDI-II (for 18–25 years) self-report symptoms scales. The CDI has 27-items with 3 response categories (0–2, total score 0–54; Timbremont et al., 2008). The BDI-II has 21-items with 4 response categories (0–3, total score 0–63; Van der Does, 2002). Internal consistency at baseline and the three follow-up assessments was .70, .77, .79, and .81, respectively, for the CDI, and .54, .83, .81, and .84, respectively, for the BDI-II. Clinical depression was established from a psychiatric interview and severity rating scales (as described above in the assessment procedure).

*Health-related quality of life* (including social functioning) was assessed with the self-report questionnaires IMPACT-III (10–20 years) and the Inflammatory Bowel Disease Questionnaire (IBDQ; 21–25 years). The IMPACT-III has 35 items, scored 1–5 (total score 35–175; Otley et al., 2002). The IBDQ contains 32 items, scored 1–5 (total score 32–160; De Boer, Wijker, Bartelsman, & de Haes, 1995). For both instruments, a higher score indicates better HRQOL. We included in the analyses the total scores and the individual subscale scores for social functioning of both instruments. For the total score, internal consistency at baseline and the three follow-up assessments was .71, .92, .90, and .90, respectively, for the IMPACT-III, and .71, .92, .85, and .88, respectively, for the IBDQ. For the social functioning subscale score, internal consistency at baseline and the three follow-up assessments was .67, .54, .59, and .49, respectively, for the IMPACT-III subscale, and .69, .85, .48, and .51, respectively, for the IBDQ subscale.

*Coping* was assessed using the Cognitive Emotion Regulation Questionnaire (CERQ). The CERQ contains 36 items, scored 1–5, subdivided into nine subscales. These scales are divided in two domains: adaptive coping (e.g., positive reappraisal) and maladaptive coping (e.g., self-blame and catastrophizing). A higher score indicates more use of a particular coping style (Garnefski, Legerstee, Kraaij, Van Den Kommer, & Teerds, 2002). Internal consistency at baseline and the three follow-up assessments was .89, .91, .94, and .94, respectively, for the adaptive coping domain, and .87, .88, .87, and .86, respectively, for the maladaptive coping domain.

*Illness perceptions* were assessed with the Brief Illness Perceptions Questionnaire (B-IPQ; Broadbent, Petrie, Main, & Weinman, 2006; De Raaij, Schröder, Maissan, Pool, & Wittink, 2012). It contains nine self-report items on cognitive and emotional representations of illness. Eight dimensions (e.g., consequences of illness, personal control, concerns, and understanding) are scored from 0 to 10. A higher score represents more negative illness perceptions. Internal consistency at baseline and the three follow-up assessments was .74, .79, .78, and .75.

*Sleep problems* were assessed using the sleep problem items of the Youth Self-Report (YSR; for ages 10–17; Achenbach and Rescorla, 2001) and the Adults Self-Report (ASR; for ages 18–25; Achenbach and Rescorla, 2003). These questionnaires contain three comparable items on sleep problems (scored 0, 1, or 2), of which the scores were added up: ‘I sleep more than most other people during day and/or night.’ and ‘I have trouble sleeping.’

*Clinical disease activity* was assessed with four validated clinical disease activity measures around the moments that the online questionnaires on psychological symptoms were filled out. For youth with CD in the age category 10–20, the short Pediatric Crohn’s Disease Activity Index (sPCDAI; Kappelman et al., 2011) was used; for those with UC and IBD-U the Pediatric Ulcerative Colitis Activity Index (PUCAI; Turner et al., 2007). For youth with CD in the age category 21–25 years, the Crohn’s Disease Activity Index (CDAI; Best, Becktel, Singleton, & Kern, 1976) was used; for those with UC and IBD-U the partial Mayo score (Schroeder, Tremaine, & Ilstrup, 1987). All are physician-rated forms (not online), which provide four categories of clinical disease activity: remission, mild, moderate, and severe.

### Statistical Analysis

We tested differences in demographic and disease characteristics between the two groups at baseline using t-tests, Mann–Whitney tests, and Chi-square tests.

To enable combining data of all participants in one analysis (thereby maximizing power), despite the use of age-appropriate instruments, we calculated a Reliable Change Index (RCI; Jacobson and Truax, 1991) value separately for anxiety and depression (primary outcomes) for each participant, at each assessment. The RCI of an instrument is calculated from the standard error of measurement (SEM) of the pretest reliability and the test–retest reliability. The RCI can have three possible values: reliably improved; no reliable change; and reliably deteriorated (see Appendix 2 for RCI details). Chi-square tests were used to test for differences in RCI values between the two groups. These analyses included only youth for whom pre- and posttest data were available (see Table 1 for the details on sample sizes for each Chi-square test). The proportions of patients who developed clinical anxiety and/or depression were compared between groups using a separate Chi-square test.

For exploratory analyses, we used linear mixed models (taking into account missing data) to compare the change on full-range scores from baseline to 6- and 12-month follow-up between groups. The outcomes were anxiety (SCARED or HADS-A), depression (CDI or BDI-II), HRQOL (IMPACT-III or IBDQ), social functioning (subscale of IMPACT-III or IBDQ), coping (CERQ), illness perceptions (B-IPQ), and sleep problems (YSR or ASR). The starting model for all outcomes included a random intercept and fixed factors for time, group, and the interaction between time and group. Next, we examined with the use of likelihood-ratio tests whether adding a random slope of time and a quadratic term of time and the interaction between the quadratic term of time with group improved the model. The restricted maximum likelihood method was applied, as this is preferred for relatively small sample sizes (Luke, 2017; Serrano, 2008). Because we had no expectations about the relationship between the random intercept and slope, an unstructured covariance structure was selected, which is the most flexible structure.

Follow-up data were analyzed based on the intention-to-treat principle, unless otherwise specified. For the Chi-square analyses (with the primary dichotomous outcomes), this implied inclusion of data of only those randomized for whom follow-up data were available (since follow-up data were required to calculate the RCI). For the exploratory analyses (secondary continuous outcomes), the intention-to-treat principle implied inclusion of data of all randomized participants, also those without follow-up data (since the linear mixed models take into account missing data and follow-up data were not required). A *p* value of < .05 was considered statistically significant. Data were analyzed using SPSS version 24.

### Sample Size and Power

Sample size and power were based on anxiety and depressive symptoms as primary outcomes. Meta-analytic studies in youth without a somatic disease have shown medium-to-large effect sizes for anxiety symptoms (Reynolds, Wilson, Austin, & Hooper, 2012) and medium effect sizes for depressive symptoms (Weisz, McCarty, & Valeri, 2006). These correspond with *φ* > 0.40 and *φ* > 0.30, for anxiety and depressive symptoms, respectively. For the main Chi-square analyses, this means that a sample size of 70 provides enough power for the anxiety outcomes (> 85%, *β* = 0.14) and medium power for the depression outcomes (> 60%, *β* = 0.39).

## Results

### Demographic Data

**Fig. 1:**
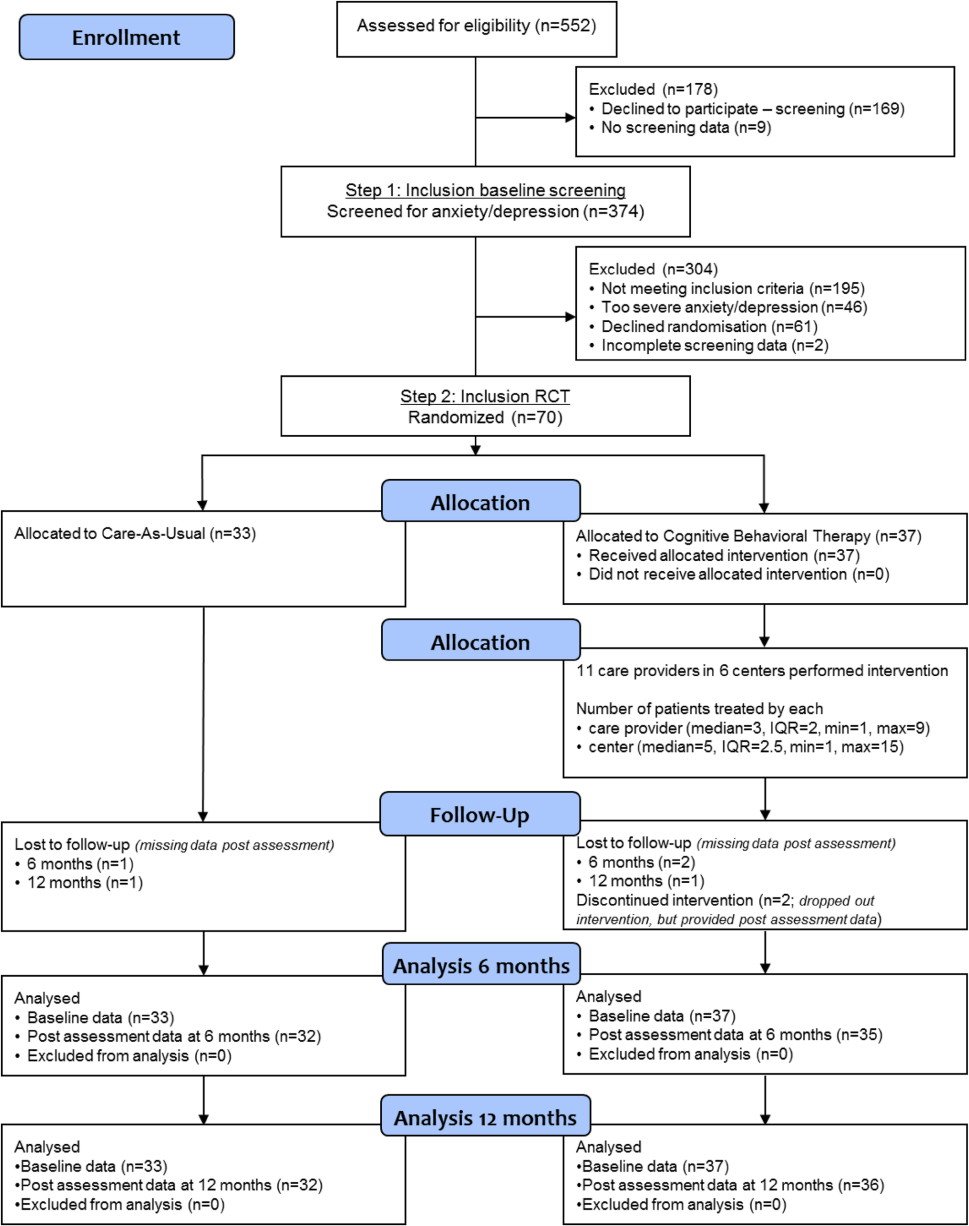
CONSORT study flow chart

In total, 70 youth were randomized; 37 in the PASCET-PI group and 33 in the CAU group (see Fig. 1). Attrition was very low; only two patients dropped out of the PASCET-PI, and only three patients (6 months) and two patients (12 months) did not complete follow-up assessments. Demographic variables did not significantly differ between the groups (see Appendix 3, Table 4): percentage of males (27.0% vs. 36.4%, *p* = .401), mean age (18.62 vs. 17.69, *p* = .393), socioeconomic status (*p* = .348), and ethnicity (*p* = .749). The numbers of patients included at baseline based on anxiety, depression, or both did not differ between the groups as well (*p* = .070). The patients’ disease characteristics did not differ between the groups: IBD subtype (% CD 48.6% vs. 54.5%), Paris classification at diagnosis (CD location; *p* = .808, CD behavior; *p* = .243, UC extent; *p* = .069, UC severity; *p* = .104), percentage of patients in clinical remission (73.0% vs. 78.8%, *p* = .571), and use of IBD medication (% immunomodulators 43.2% vs. 48.5% and % biologicals 21.6% vs. 36.4%). However, the median disease duration was longer in the PASCET-PI group than in the CAU group (2.59 vs. 1.17 years, *p* = .039). In the PASCET-PI group, 18 patients were aged 10–17 years and 19 patients 18–25 years. In the CAU group, 17 patients were aged 10–17 years and 16 patients 18–25 years.

With respect to treatment integrity, adherence to the protocol was good. The mean number of sessions followed in the PASCET-PI group was 9.38 (out of 10). The mean number of family sessions followed was 2.57 (out of 3), and the mean number of booster sessions followed was 2.59 (out of 3). In all sessions, at least 75% of the topics were discussed.

### Effect of Disease-Specific CBT on Symptoms of Depression and Anxiety

In the Chi-square tests, some cells in the crosstabulation were smaller than 5. Only few patients (0–4) were in the ‘Reliable increase of score/deterioration’ category (i.e., deteriorated in anxiety or depression). Therefore, we combined this category with the ‘No reliable change’ category, to test if the PASCET-PI and CAU groups differed with respect to the proportions of patients who had improved on anxiety or depression. In these main analyses, RCI values for anxiety [*χ*^2^ (1) = .226, *p* = .801] and depression [*χ*^2^ (1) = 2.680, *p* = .141] after 6 months did not differ between the groups, neither did the RCI values for anxiety [*χ*^2^ (1) = .337, *p* = .626] after 12 months. This indicates that in both groups a similar proportion of patients had improved. For depression after 12 months, the RCI values differed significantly between the groups, indicating that the proportion of patients in the CAU group that had improved was higher than that in the PASCET-PI group [*χ*^2^ (1) = 5.460, *p* = .026], see Table 1. In the PASCET-PI group, two patients developed clinical anxiety and/or depression during follow-up, versus one patient in the CAU group, which was not significantly different [*χ*^2^ (1) = .240, *p* = .543].

**Table 1:** Crosstabulation of 6- and 12-month RCI of symptoms of anxiety and depression versus group

	Reliable increase of score/deterioration or no reliable change	Reliable decrease of score/improvement	Total
*6* *months*			
RCI categories anxiety (SCARED or HADS-A)^a^			
CAU	11 (34.4%)	21 (65.6%)	32
CBT	14 (40.0%)	21 (60.0%)	35
RCI categories depression (CDI or BDI-II)^b^			
CAU	11 (34.4%)	21 (65.6%)	32
CBT	19 (54.3%)	16 (45.7%)	35
*12* *months*			
RCI categories anxiety (SCARED or HADS-A)^c^			
CAU	12 (37.5%)	20 (62.5%)	32
CBT	16 (44.4%)	20 (55.6%)	36
RCI categories depression (CDI or BDI-II)^d^			
CAU	8 (25.0%)	24 (75%)	32
CBT	19 (52.8%)	17 (47.2%)	36

^a^Pearson Chi-square = .226, *p* = .801, *φ* = − .058 (95%BI .000–.293). Numbers in parentheses indicate row percentages

^b^Pearson Chi-square = 2.680, *p* = .141, *φ* = .200 (95%BI .000–.439). Numbers in parentheses indicate row percentages

^c^Pearson Chi-square = .337, *p* = .626, *φ* = .070 (95%BI .000–.306). Numbers in parentheses indicate row percentages

^d^Pearson Chi-square = 5.460, *p* = .026, *φ* = .283 (95%BI .036–.521). Numbers in parentheses indicate row percentages

To provide more insight into age differences, we also performed the Chi-square analyses separately for the 10- to 17-year olds and the 18- to 25-year olds. The results almost completely matched those from the Chi-square analyses in the total group (data not shown). However, the proportion of 18- to 25-year olds in the CAU group that had improved on depression after 12 months was higher than that in the PASCET-PI group [*χ*^2^ (1) = 6.349, *p* = .019].

The exploratory analyses gave similar results as the Chi-square analyses. For all outcomes, the residuals of the models were approximately normally distributed. For the SCARED and the IMPACT-III, the final model included a fixed factor for time and group, a random intercept, and a random slope for time, since the likelihood-ratio test indicated that adding a random slope for time improved the model significantly. Because this was not the case for all the other outcomes, the respective models did not include a random slope for time. For the BDI-II, the final model included fixed factors for time and group, and for the interaction between time and group, and a random intercept. For all the other outcomes, however, including a fixed factor for the interaction between time and group did not improve the model. Therefore, for all other outcomes, the final model included fixed factors for time and group, and a random intercept. For the SCARED, HADS-A, and BDI-II, adding a quadratic term of time significantly improved the model. Then adding the interaction between the quadratic terms of time and group did not improve the model for these outcomes.

A significant time–group (PASCET-PI versus CAU group) interaction effect was not found for anxiety (SCARED: *p* = .798; HADS-A: *p* = .997), depression (CDI: *p* = .693), HRQOL (IMPACT-III total score: *p* = .117; IBDQ total score: *p* = .247), social functioning (IMPACT-III Social functioning: *p* = .407; IBDQ Social functioning; *p* = .879), coping (CERQ Adaptive coping: *p* = .506; CERQ Maladaptive coping: *p* = .592), illness perceptions (B-IPQ: *p* = .474), and sleep problems (YSR/ASR: *p* = .858). The only significant time–group interaction was found on the BDI-II (*p* = .025; favoring the CAU group over the course of 12 months). Therefore, for all outcomes except the BDI-II, the effect of time was similar for both groups. Table 2 presents the coefficients for time presented for the model without the interaction of time and group; only for the BDI-II the estimate is presented for the model with the interaction of time and group included. Cohen’s *d* reflects the effect size for the overall means, since no group differences were observed, except for the BDI-II. For all outcomes (except sleep problems; *p* = .070), the average effect of time was significant, indicating that over the course of 12 months, youth in both groups had improved on their psychological outcomes (anxiety, depression, HRQOL, social functioning, coping, and illness perceptions). For the SCARED, HADS-A, and BDI-II, the quadratic effect was significant. This indicates that for these outcomes the model follows a quadratic trajectory over the course of 12 months.

**Table 2:** Results of linear mixed models: time effects for outcome variables with overall Estimated Marginal Means

Variable	*β* (SE) (time effect)^a^	*p* (time effect)	*β* (SE) (time^2^ effect)^a^	*p* (time^2^ effect)	Baseline Mean (SE)	6 Months Mean (SE)	Cohen’s *d* (baseline—6 months)	12 Months Mean (SE)	Cohen’s *d* (baseline—12 months)
SCARED^b,c^ (anxiety; 10–20 years, n = 50)	− 1.065 (.103)	**<.001**	.013 (.002)	**<.001**	37.8 (1.9)	18.7 (1.9)	− 1.41	18.6 (2.3)	− 1.27
HADS-A^c^ (anxiety; 21–25 years, n = 20)	− .216 (.037)	**<.001**	.003 (.001)	**<.001**	9.5 (0.6)	5.9 (0.6)	− 1.39	6.3 (0.6)	− 1.14
CDI (depression; 10–17 years, n = 35)	− .078 (.013)	**<.001**	NA	NA	9.0 (0.8)	6.9 (0.7)	− 0.71	4.9 (0.8)	− 1.01
BDI-II^c^ (depression; 18–25 years, n = 35)	− .360 (.057)^d^	**<.001**	.005 (.001)	**<.001**	13.9 (1.2)	5.8 (1.1)	− 1.82^e^	5.2 (1.2)	− 1.81^e^
IMPACT-III total score^b^ (HRQOL; 10–20 years, n = 50)	.223 (.035)	**<.001**	NA	NA	140.1 (2.0)	146.1 (1.8)	0,82	151.9 (2.1)	0.84
IMPACT-III Social functioning (10–20 years, n = 50)	.055 (.013)	**<.001**	NA	NA	49.5 (0.8)	51.0 (0.7)	0.47	52.4 (0.8)	0.53
IBDQ total score (HRQOL; 21–25 years, n = 20)	.292 (.094)	**.003**	NA	NA	168.1 (3.8)	176.0 (3.1)	1.02	183.5 (4.2)	0.84
IBDQ social functioning (21–25 years, n = 20)	.060 (.029)	**.006**	NA	NA	29.7 (0.9)	31.3 (0.8)	0.58	32.9 (1.0)	0.71
CERQ adaptive coping (10–25 years, n = 70)	− .086 (.037)	**.024**	NA	NA	59.1 (1.8)	56.8 (1.7)	− 0.21	54.6 (2.1)	− 0.31
CERQ maladaptive coping (10–25 years, n = 70)	− .092 (.020)	**<.001**	NA	NA	27.8 (0.9)	25.3 (0.8)	− 0.50	22.9 (1.1)	− 0.68
B-IPQ (illness perceptions; 10–25 years, n = 70)	− .149 (.022)	**<.001**	NA	NA	39.9 (1.3)	35.9 (1.2)	0.55	32.0 (1.4)	− 0.71
YSR/ASR (sleep problems; 10–25 years, n = 70)	− .004 (.003)	.070	NA	NA	0.8 (0.1)	0.7 (0.1)	0.24	0.6 (0.1)	− 0.26

Significant effects are highlighted in bold

*NA* not applicable

^a^For the SCARED, HADS-A, CDI, BDI-II, CERQ Adaptive coping, B-IPQ, and YSR/ASR, a negative beta indicates improvement of problems and a negative Cohen’s *d* indicates improvement of problems over time. For the IMPACT-III, IMPACT-III Social functioning, IBDQ, IBDQ Social functioning, and CERQ maladaptive coping, a positive beta indicates improvement of problems and a positive Cohen’s *d* indicates improvement of problems over time. For all outcomes, the beta is the time effect for both groups, unless otherwise specified

^b^For these outcomes, the linear mixed model also included a random slope for time, whereas for all the other outcomes the model included only fixed factors and a random intercept

^c^For these outcomes, the linear mixed model also included a quadratic term of time

^d^Since the interaction of time and group is significant for the BDI-II, this beta is the time effect for the control group

^e^For the BDI-II, Cohen’s *d* reflects the effect size for the control group

## Discussion

In the current RCT, we examined the long-term effects of a disease-specific CBT on psychological outcomes of youth with IBD. Overall, youth in both groups improved on anxiety and depressive symptoms, HRQOL, social functioning, coping, and illness perceptions and these improvements sustained until the final follow-up assessment at 12 months. In both groups, a similar proportion of patients improved in anxiety and/or depression (main analyses) and the groups did not differ in the proportion of patients that developed clinical anxiety and/or depression. However, in general, no differences between the CBT and CAU groups were found.

Our results are partly in line with the results of similar trials. Levy et al. (2016) found that three sessions of SLCBT outperformed educational support, but only in improving HRQOL (after 1 week of follow-up), coping and school attendance (after 12 months of follow-up), and in parent- and child-reported distract/ignore coping of the child. In line with our results, SLCBT did not have a beneficial effect on anxiety, depression, coping, or functional disability. Szigethy et al. (2014) found that CBT outperformed supportive non-directive therapy in improving disease activity after three months, with a difference of 10 points in raw disease activity scores from pre- to post-intervention. When only data of patients with active CD were analyzed, CBT was more effective than was supportive non-directive therapy in improving disease activity and somatic depressive symptoms after three months of treatment (Szigethy et al., 2015).

Explanations for the lack of an effect of the disease-specific CBT in our trial may be the following. First, most of the participants had no or only mild somatic symptoms at baseline, reflected by low IBD disease activity scores. Receiving the full protocol of CBT may have been “over-treatment” in patients with a rather low burden of disease, somatically as well as psychologically. Still, many participants remarked that the acquired skills would be useful and necessary in times of disease exacerbations. Thus, we hypothesize that CBT may be more useful for youth with severe anxiety/depression and/or those with active disease.

Second, participants in the control group may—for the very reason that they participated in a trial– have received more than just standard medical care. Via the informed consent form and the invitation by the medical staff, they were informed about psychological problems in IBD. Then, they were systematically screened with questionnaires and diagnostic interviews. This provided them with the opportunity to express their emotions and concerns, which may have evoked feelings of reassurance and safety. The created awareness may have benefitted all patients, and may have been enough to improve subclinical anxiety and depression. This also may explain why so few patients in both groups developed clinical anxiety and/or depression.

It is unexpected and counterintuitive that at 12 months of follow-up the proportion of patients that had improved on depressive symptoms was the highest in the CAU group, although this only held for the 18- to 25-year olds. Youth in this age range have a more advanced cognitive development than younger peers. Those receiving CBT may find themselves confronted with the life-long impact of IBD on, for example, long-lasting romantic relationships, work, and career prospects. This may maintain the depressive symptoms. Still, this unexpected finding, may have been a chance finding, considering the number of statistical tests and also considering that at 3 and 6 months no difference in depression was found between the CBT and CAU groups.

Furthermore, at baseline, the disease duration in the CBT group had been significantly longer than that in the CAU group (2.59 vs. 1.17 years). This may have had an effect on the outcomes, since a shorter disease duration has been associated with lower HRQOL or more emotional/behavioral problems (Hill et al., 2010; Mackner and Crandall, 2005). It is not likely, however, that participants in the CBT group may have had fewer psychological problems, and, therefore, less room to improve considering the fact that both the RCI analysis and the exploratory linear mixed models took into account the baseline psychological outcomes scores.

Since the PASCET-PI was originally developed and found effective for improving mainly depression (Szigethy et al., 2014), it was unexpected that we found no differences on depressive symptoms. Furthermore, we found no additional effect on anxiety symptoms, although we adapted the PASCET-PI to also target anxiety. Szigethy et al. (2015) only found an additional effect of CBT on somatic depressive symptoms and disease activity in patients with active CD. In our study, approximately three-quarters of the participants were in clinical remission, which may explain differences in results.

We also did not find differences between the groups in improvement in coping and negative illness perceptions. Changes in coping or illness perceptions after psychological treatment can only be expected if the treatment had focused specifically on these factors. The PASCET-PI contains components that may influence coping (e.g., practicing positive thinking) and illness perceptions (e.g., discussing the illness narrative). Perhaps, the current protocol had too little focus on challenging coping styles in the current protocol. An alternative explanation may be the low level of negative illness perceptions, on account of low disease activity (e.g., Schröder et al., 2012; Busscher and Spinhoven, 2017). However, the secondary analyses were exploratory (and conducted in subgroups of patients based on age). As a consequence, the study design may not have been the most suitable to investigate coping and illness perceptions. Future studies should therefore investigate how coping and illness perceptions can be the focus of psychological treatment to improve anxiety and depression.

### Clinical and Future Directions

Considering the results of the current study and that of earlier studies into CBT for youth with IBD (Szigethy et al., 2014, 2015; Levy et al., 2016), it remains unclear which patients with IBD will benefit the most from CBT, how the intervention should be delivered, and what outcomes improve the most.

From our findings we conclude that providing a full protocol of disease-specific CBT for preventive purposes seems not necessary. We assume that that patients with more clinical anxiety and/or depression likely will benefit more from CBT, as was found in both youth (Szigethy et al., 2014) and adults with IBD (Bennebroek Evertsz et al., 2017). Moreover, although this is not clear yet, a full protocol of CBT may be more helpful for improving both psychological and somatic symptoms of IBD patients who suffer from active disease. However, since these patients are often hospitalized or need intensive pharmacological treatment, the question arises how the CBT can be delivered best to them (e.g., via telephone or Internet). Group interventions in the hospital have been shown to be promising in youth with IBD (Grootenhuis, Maurice-Stam, Derkx, & Last, 2009) and effective in youth with chronic illnesses (including IBD; Scholten et al., 2013). Furthermore, apart from anxiety, depression, and HRQOL, other clinically relevant psychological outcomes such as social functioning, school attendance, or treatment adherence may be important to target as well. Psychological interventions aiming at these outcomes have been shown to be effective in youth with either IBD or other chronic illnesses (Forgeron, King, Reszel, & Fournier, 2017; Hommel et al., 2012).

### Strengths and Limitations

One of the strengths of the current study is the randomized and prospective design, in which the interviewer and the treating physicians were blinded to the group assignment. In addition, we included patients with a broad and clinical relevant age range and our findings have external validity since patients came from both rural and urban centers (including different therapists). Furthermore, the study had very low attrition and we investigated several psychological outcomes. An important limitation is that we did not control for attention placebo effects. We chose to use standard medical care as control condition, because it was already known that CBT as state-of-the-art psychotherapy performs better than placebo for anxiety and depression. We deemed this the most clinical relevant comparison, considering that this best resembles our current care. Furthermore, the relatively small sample size is a limitation, although the study was sufficiently powered. In addition, to cover the whole age range, so that the exploratory linear mixed models could only be performed on subgroups.

## Conclusion

Overall, youth in both the CBT and the control group improved on their psychological outcomes 6 and 12 months after baseline. CBT did not have an additional effect in improving anxiety, depression, HRQOL, social functioning, coping, illness perceptions, and sleep problems, when compared to the control condition. We think that the awareness created by participating in a RCT had a positive effect on the psychological outcomes of youth in both groups. Offering a full protocol of CBT in youth with relatively low somatic and psychological burden does not seem to be necessary.

## Figures and Tables

**Figure:**
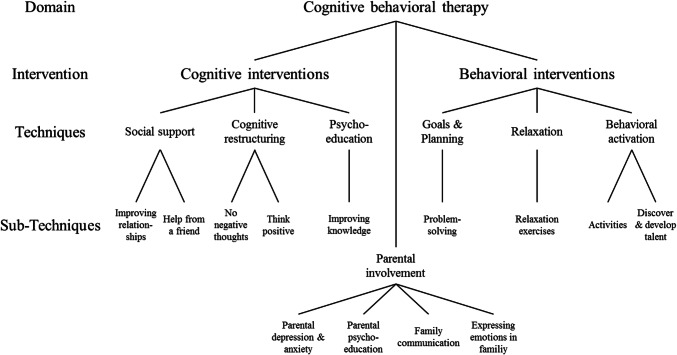
Tree diagram PASCET-PI

## Appendix 1

### Outline and Tree Diagram of the PASCET-PI

See Table 3.

**Table 3:** Outline of the PASCET-PI (Szigethy et al., 2007; van den Brink et al., 2016)

Session number	Content of session
Session 1 *Live (60 min)*	Introduction of **ACT** & **THINK** model and PASCET-PI, building work alliance, psycho-education about IBD and depression or anxiety, illness narrative
Session 2 *Live (60 min)*	Mood monitoring, explaining link between feelings, thoughts, and behaviors, discussing feeling good and feeling bad, problem-solving
Session 3 *By telephone (30 min)*	Link between behavior and feelings: **A**ctivities to feel better
Session 4 *Live (60 min)*	Be **C****alm** and Confident: relaxation exercises
Session 5 *Live (60 min)*	Be Calm and **C****onfident**: positive self versus negative self, training social skills
Session 6 *By telephone (30 min)*	**T**alents: developing talents and skills makes you feel better
Session 7 *Live (60 min)*	Social problem-solving, discussing the **ACT** skills and introduction of the **THINK** skills with discussing negative thoughts (**T**hink positive/cognitive restructuring)
Session 8 *By telephone (30 min)*	**H**elp from a friend, **I**dentify the ‘Silver Lining,’ and **N**o replaying bad thoughts
Session 9 *By telephone (30 min)*	**K**eep trying—Don’t give up, making several plans to use the **ACT** & **THINK** skills
Session 10 *Live (60 min)*	Quiz on **ACT** & **THINK** model, discussing use of **ACT** & **THINK** skills in the future, updating illness narrative
Booster 1 *By telephone (30 min)*	Several plans to use the **ACT** & **THINK** skills, updating illness narrative, personalizing **ACT** & **THINK** skills
Booster 2 *By telephone (30 min)*	Several plans to use the **ACT** & **THINK** skills, updating illness narrative, personalizing **ACT** & **THINK** skills
Booster 3 *By telephone (30 min)*	Several plans to use the **ACT** & **THINK** skills, updating illness narrative, personalizing **ACT** & **THINK** skills
Family 1 *Live (60 min)*	Parental view on IBD, family situation, psycho-education about IBD and depression or anxiety, introduction of **ACT** & **THINK** model and PASCET-PI
Family 2 *Live (60 min)*	Parental view on progress, the **ACT** & **THINK** skills that are most effective for the patient, expressing emotions within family, family communication, family stress game
Family 3 *Live (60 min)*	Parental view on progress, family communication, parental depression, or anxiety

*IBD* inflammatory bowel disease, *PASCET-PI* primary and secondary control enhancement training for physical illness

## Appendix 2

### Calculation of Reliable Change Index (RCI) Variables

- *Step 1* Calculating the standard error of difference for each participant, separately for anxiety and depression:
  $${\text{RC}} = \frac{{x_{2} - x_{1} }}{{S_{\text{diff}} }}\;\;\;\;S_{\text{diff}} = \sqrt {2({\text{SE}})^{2} } \;\;\;\;S_{\text{E}} = S_{1} \sqrt {1 - r_{xx} },$$
- in which *x*_1_ and *x*_2_ are the individual’s scores on baseline and at follow-up, respectively; *S*_1_ is the pretest variance for that instrument; and *r*_*xx*_ is the test–retest reliability of the instrument as reported in the manual.
  - SCARED (10–20 years): *r*_*xx*_= .81 (Muris et al., 2011) |* S*_1_ = 13.389→|* S*_diff_ = 8.253
  - HADS-A (21–25 years): *r*_*xx*_= .89 (Spinhoven et al., 1997) |* S*_1_ = 2.373→|* S*_diff_ = 1.113
  - CDI (10–17 years): *r*_*xx*_= .86 (Timbremont et al., 2008) |* S*_1_ = 4.648→|* S*_diff_ = 2.459
  - BDI-II (18–25 years): *r*_*xx*_= .93 (Van der Does, 2002) |* S*_1_ = 4.381→|* S*_diff_ = 1.639
- *Step 2* Calculating the difference between the follow-up and the baseline for each participant, separately for anxiety and depression.
- *Step 3* Calculating the RC value for each participant, separately for anxiety and depression.
- *Step 4* Determining the RCI value for each participant, separately for anxiety and depression. Both for anxiety and depression this leads to a variable with three possible values: no reliable change, reliable deterioration, and reliable improvement. An RC value of between − 1.96 and 1.96 indicates no reliable change (*p *< .05). When RC is higher than 1.96, this indicates a reliable increase in the score (*p *< .05), i.e., reliable deterioration (as for all the instruments applies that a higher score represents more symptoms). When RC is lower than − 1.96, this indicates a reliable decrease in the score (*p *< .05), i.e., reliable improvement.

## Appendix 3

See Table 4.

**Table 4:** Baseline demographic and disease characteristics

	PASCET-PI group (*n *= 37)	CAU group (*n *= 33)	*p* value
Demographic status			
Male, *n* (%)	10 (27.0)	12 (36.4)	.401^a^
Age, mean (SD), years	18.62 (4.27)	17.69 (4.82)	.393^b^
SES, *n* (%)			
Low	8 (21.6)	4 (12.9)	.348^a^
Middle	15 (40.5)	10 (32.3)	
High	14 (37.8)	17 (54.8)	
Ethnicity, *n* (%) (*n* = 64)			
Dutch/Western	30 (81.1)	25 (80.6)	.749^a^
Other	7 (18.9)	6 (19.4)	
Included on, *n* (%)			
Anxiety	30 (81.1)	20 (60.6)	.070^a^
Depression	0 (0.0)	3 (9.1)	
Both	7 (18.9)	10 (30.3)	
IBD subtype, *n* (%)			
Crohn’s disease	18 (48.6)	18 (54.5)	.808^a^
Ulcerative colitis	14 (37.8)	12 (36.4)	
IBD-U	5 (13.5)	3 (9.1)	
Paris classification at diagnosis, *n* (%)			
CD: location (*n* = 36)^†^			
L1	4 (22.2)	2 (11.1)	.813^a^
L2	4 (22.2)	4 (22.2)	
L3	6 (33.3)	8 (44.4)	
+ L4a/L4b	4 (22.2)	4 (22.2)	
CD: behavior (*n* = 36)			
Non-stricturing, non-penetrating	18 (100.0)	16 (88.9)	.243^c^
Stricturing, penetrating, or both	0 (0.0)	2 (11.1)	
UC: extent (*n* = 34)^‡^			
Limited: E1 + E2	11 (57.9)	4 (26.7)	.069^a^
Extensive: E3 + E4	8 (42.1)	11 (73.3)	
UC: severity			
Never severe	18 (94.7)	11 (73.3)	.104^c^
Ever severe	1 (5.3)	4 (26.7)	
Clinical disease activity, *n* (%)			
Remission	27 (73.0)	26 (78.8)	.571^a^
Mild	10 (27.0)	7 (21.2)	
Disease duration, median, years	2.59	1.17	.039^d^
IBD Medications, *n* (%)			
Aminosalicylates	18 (48.6)	12 (36.4)	.300^a^
Immunomodulators	16 (43.2)	16 (48.5)	.660^a^
Biologicals	8 (21.6)	12 (36.4)	.173^a^
Corticosteroids^§^	2 (5.4)	5 (15.2)	.170^c^
Enemas	3 (8.1)	1 (3.0)	.352^c^
No medication	2 (5.4)	1 (3.0)	.543^c^

*PASCET-PI* primary and secondary control enhancement training for physical illness, *CAU* care-as-usual, *SD* standard deviation, *IBD* inflammatory bowel disease, *IBD-U* inflammatory bowel disease unclassified, *SES* socioeconomic status

^a^Chi-square

^b^ANOVA

^c^Fisher’s Exact test

^d^Mann–Whitney test | *UC includes IBD-U patients, ^†^L1: ileocecal, L2: colonic, L3: ileocolonic, L4a: upper gastrointestinal tract proximal, and L4b distal from Treitz ligament ^‡^E1: proctitis, E2: left-sided colitis distal of splenic flexure, E3: extensive colitis distal of hepatic flexure, E4: pancolitis ^§^prednisone (oral and intravenous) and budesonide (oral)

## References

[CR1] Achenbach TM, Rescorla LA (2001). Manual for the ASEBA school-age forms & profiles.

[CR2] Achenbach TM, Rescorla LA (2003). Manual for the ASEBA adult forms & profiles.

[CR3] Adamiak, T., Walkiewicz-Jedrzejczak, D., Fish, D., Brown, C., Tung, J., Khan, K., … Kugathasan, S. (2013). Incidence, clinical characteristics, and natural history of pediatric IBD in Wisconsin: a population-based epidemiological study. *Inflammatory Bowel Diseases, 19*, 1218–1223. 10.1097/mib.0b013e318280b13e. 10.1097/MIB.0b013e318280b13e PMC4898969 23528339

[CR4] Arnett JJ (2010). Adolescence and emerging adulthood: A cultural approach (International Edition).

[CR5] Bennebroek Evertsz, F., Sprangers, M. A. G., Sitnikova, K., Stokkers, P. C. F., Ponsioen, C. Y., Bartelsman, J., … Bockting, C. L. H. (2017). Effectiveness of cognitive-behavioral therapy on quality of life, anxiety, and depressive symptoms among patients with inflammatory bowel disease: A multicenter randomized controlled trial. *Journal of Consulting and Clinical Psychology, 85*, 918–925. 10.1037/ccp0000227 28857595

[CR6] Best WR, Becktel JM, Singleton JW, Kern F (1976). Development of a Crohn’s disease activity index. National Cooperative Crohn’s Disease Study. Gastroenterology.

[CR7] Bodden DHM, Bögels SM, Muris P (2009). The diagnostic utility of the Screen for Child Anxiety Related Emotional Disorders-71 (SCARED-71). Behaviour Research and Therapy.

[CR8] Bonaz BL, Bernstein CN (2013). Brain-gut interactions in inflammatory bowel disease. Gastroenterology.

[CR9] Boutron I, Altman DG, Moher D, Schulz KF, Ravaud P, Group, C. N. (2017). CONSORT Statement for Randomized Trials of Nonpharmacologic Treatments: A 2017 update and a CONSORT extension for nonpharmacologic trial abstracts. Annals of Internal Medicine.

[CR10] Broadbent E, Petrie KJ, Main J, Weinman J (2006). The brief illness perception questionnaire. Journal of Psychosomatic Research.

[CR11] Brooks AJ, Rowse G, Ryder A, Peach EJ, Corfe BM, Lobo AJ (2016). Systematic review: Psychological morbidity in young people with inflammatory bowel disease—Risk factors and impacts. Alimentary Pharmacology & Therapeutics.

[CR12] Busscher B, Spinhoven P (2017). Cognitive coping as a mechanism of change in cognitive-behavioral therapy for fear of flying: A longitudinal study with 3-year follow-up. Journal of Clinical Psychology.

[CR13] Christensen SS, Frostholm L, Ornbol E, Schroder A (2015). Changes in illness perceptions mediated the effect of cognitive behavioural therapy in severe functional somatic syndromes. Journal of Psychosomatic Research.

[CR15] De Boer AG, Wijker W, Bartelsman JF, de Haes HC (1995). Inflammatory Bowel Disease Questionnaire: Cross-cultural adaptation and further validation. European Journal of Gastroenterology and Hepatology.

[CR16] De Croon, E. M., Nieuwenhuijsen, K., Hugenholtz, N. I. R., & Van Dijk, F. J. H. (2005). Drie vragenlijsten voor diagnostiek van depressie en angststoornissen. *TBV–Tijdschrift voor Bedrijfs-en Verzekeringsgeneeskunde, 13*, 114–119.

[CR17] De Raaij EJ, Schröder C, Maissan FJ, Pool JJ, Wittink H (2012). Cross-cultural adaptation and measurement properties of the Brief Illness Perception Questionnaire-Dutch Language Version. Manual Therapy.

[CR18] Forgeron P, King S, Reszel J, Fournier K (2017). Psychosocial interventions to improve social functioning of children and adolescents with chronic physical conditions: A systematic review. Children’s Health Care.

[CR19] Garnefski N, Legerstee J, Kraaij V, Van Den Kommer T, Teerds JAN (2002). Cognitive coping strategies and symptoms of depression and anxiety: A comparison between adolescents and adults. Journal of Adolescence.

[CR20] Ginsburg G, Keeton C, Drazdowski T, Riddle M (2011). The utility of clinicians ratings of anxiety using the pediatric anxiety rating scale (PARS). Child & Youth Care Forum.

[CR21] Gracie DJ, Guthrie EA, Hamlin PJ, Ford AC (2018). Bi-directionality of brain-gut interactions in patients with inflammatory bowel disease. Gastroenterology.

[CR22] Greenley RN, Hommel KA, Nebel J, Raboin T, Li SH, Simpson P, Mackner L (2010). A meta-analytic review of the psychosocial adjustment of youth with inflammatory bowel disease. Journal of Pediatric Psychology.

[CR23] Griffiths AM (2004). Specificities of inflammatory bowel disease in childhood. Best Practice & Research Clinical Gastroenterology.

[CR24] Grootenhuis MA, Maurice-Stam H, Derkx BH, Last BF (2009). Evaluation of a psychoeducational intervention for adolescents with inflammatory bowel disease. European Journal of Gastroenterology and Hepatology.

[CR25] Hamilton M (1959). The assessment of anxiety states by rating. British Journal of Medical Psychology.

[CR26] Hamilton M (1960). A rating scale for depression. Journal of Neurology, Neurosurgery and Psychiatry.

[CR27] Hill, R., Lewindon, P., Muir, R., Grange, I., Connor, F., Ee, L., … Davies, P. (2010). Quality of life in children with Crohn disease. *Journal of Pediatric Gastroenterology and Nutrition, 51*, 35–40. 10.1097/mpg.0b013e3181c2c0ef. 10.1097/MPG.0b013e3181c2c0ef 20410845

[CR28] Hommel KA, Hente EA, Odell S, Herzer M, Ingerski LM, Guilfoyle SM, Denson LA (2012). Evaluation of a group-based behavioral intervention to promote adherence in adolescents with inflammatory bowel disease. European Journal of Gastroenterology and Hepatology.

[CR29] Jacobson NS, Truax P (1991). Clinical significance: a statistical approach to defining meaningful change in psychotherapy research. Journal of Consulting and Clinical Psychology.

[CR30] Kappelman, M. D., Crandall, W. V., Colletti, R. B., Goudie, A., Leibowitz, I. H., Duffy, L., … Margolis, P. (2011). Short pediatric Crohn’s disease activity index for quality improvement and observational research. *Inflammatory Bowel Diseases, 17*, 112–117. 10.1002/ibd.21452 PMC2998542 20812330

[CR31] Kendall, P. C., Cummings, C. M., Villabo, M. A., Narayanan, M. K., Treadwell, K., Birmaher, B., … Albano, A. M. (2016). Mediators of change in the Child/Adolescent Anxiety Multimodal Treatment Study. *Journal of Consulting and Clinical Psychology, 84*, 1–14. 10.1037/a0039773 PMC4695375 26460572

[CR32] Knowles SR, Wilson JL, Connell WR, Kamm MA (2011). Preliminary examination of the relations between disease activity, illness perceptions, coping strategies, and psychological morbidity in Crohn’s disease guided by the common sense model of illness. Inflammatory Bowel Diseases.

[CR33] Levy, R. L., van Tilburg, M. A., Langer, S. L., Romano, J. M., Walker, L. S., Mancl, L. A., … Whitehead, W. E. (2016). Effects of a cognitive behavioral therapy intervention trial to improve disease outcomes in children with inflammatory bowel disease. *Inflammatory Bowel Diseases, 22*, 2134–2148. 10.1097/MIB.0000000000000881 PMC4995069 27542131

[CR34] Loftus EV, Guerin A, Yu AP, Wu EQ, Yang M, Chao J, Mulani PM (2011). Increased risks of developing anxiety and depression in young patients with crohn’s disease. American Journal of Gastroenterology.

[CR35] Luke SG (2017). Evaluating significance in linear mixed-effects models in R. Behavior Research Methods.

[CR36] Mackner LM, Crandall WV (2005). Long-term psychosocial outcomes reported by children and adolescents with inflammatory bowel disease. The American Journal of Gastroenterology.

[CR37] Manhart A-K, Hellmann S, Hamelmann E, Schlarb AA (2016). The association of sleep with inflammatory bowel disease in children and adolescents. Somnologie.

[CR38] Matza LS, Morlock R, Sexton C, Malley K, Feltner D (2010). Identifying HAM-A cutoffs for mild, moderate, and severe generalized anxiety disorder. International Journal of Methods in Psychiatric Research.

[CR39] McCombie AM, Mulder RT, Gearry RB (2013). How IBD patients cope with IBD: A systematic review. Journal of Crohn’s and Colitis.

[CR40] Mikocka-Walus A, Andrews JM, Bampton P (2016). Cognitive behavioral therapy for IBD. Inflammatory Bowel Diseases.

[CR41] Mikocka-Walus A, Pittet V, Rossel JB, von Kanel R, Swiss, IBD Cohort Study Group (2016). Symptoms of depression and anxiety are independently associated with clinical recurrence of inflammatory bowel disease. Clinical Gastroenterology and Hepatology.

[CR42] Muris P, Bodden D, Hale W, Birmaher B, Mayer B (2011). SCARED-NL. Handleiding bij de gereviseerde Nederlandse versie van de Screen for Child Anxiety Related Emotional Disorders.

[CR43] Otley A, Smith C, Nicholas D, Munk M, Avolio J, Sherman PM, Griffiths AM (2002). The IMPACT questionnaire: A valid measure of health-related quality of life in pediatric inflammatory bowel disease. Journal of Pediatric Gastroenterology and Nutrition.

[CR44] Pirinen T, Kolho K-L, Ashorn M, Aronen ET (2014). Sleep and emotional and behavioral symptoms in adolescents with inflammatory bowel disease. Sleep Disorders.

[CR45] Poznanski EO, Grossman JA, Buchsbaum Y, Banegas M, Freeman L, Gibbons R (1984). Preliminary studies of the reliability and validity of the Children’s Depression Rating Scale. Journal of the American Academy of Child Psychiatry.

[CR46] Revah-Levy A, Birmaher B, Gasquet I, Falissard B (2007). The Adolescent Depression Rating Scale (ADRS): A validation study. BMC Psychiatry.

[CR47] Reynolds S, Wilson C, Austin J, Hooper L (2012). Effects of psychotherapy for anxiety in children and adolescents: A meta-analytic review. Clinical Psychology Review.

[CR48] Rochelle TL, Fidler H (2013). The importance of illness perceptions, quality of life and psychological status in patients with ulcerative colitis and Crohn’s disease. Journal of Health Psychology.

[CR49] Rosen MJ, Dhawan A, Saeed SA (2015). Inflammatory bowel disease in children and adolescents. JAMA Pediatrics.

[CR50] Ross SC, Strachan J, Russell RK, Wilson SL (2011). Psychosocial functioning and health-related quality of life in paediatric inflammatory bowel disease. Journal of Pediatric Gastroenterology and Nutrition.

[CR51] Sauer CG, Kugathasan S (2009). Pediatric inflammatory bowel disease: Highlighting pediatric differences in IBD. Gastroenterology Clinics of North America.

[CR52] Scholten, L., Willemen, A. M., Last, B. F., Maurice-Stam, H., van Dijk, E. M., Ensink, E., … Grootenhuis, M. A. (2013). Efficacy of psychosocial group intervention for children with chronic illness and their parents. *Pediatrics, 131*, e1196–1203. 10.1542/peds.2012-2222 23478870

[CR53] Schröder A, Rehfeld E, Ørnbøl E, Sharpe M, Licht RW, Fink P (2012). Cognitive–behavioural group treatment for a range of functional somatic syndromes: Randomised trial. British Journal of Psychiatry.

[CR54] Schroeder KW, Tremaine WJ, Ilstrup DM (1987). Coated oral 5-aminosalicylic acid therapy for mildly to moderately active ulcerative colitis. A randomized study. The New England Journal of Medicine.

[CR55] Serrano D (2008). Error of estimation and sample size in the linear mixed model. (MA).

[CR56] Siebelink BM, Treffers PDA (2001). Anxiety Disorders Interview Schedule for DSM-IV-Child Version, ADIS-C Handleiding.

[CR100] Spinhoven PH, Ormel J, Sloekers PPA, Kempen GIJM, Speckens AEM, Hemert AMV (1997). A validation study of the Hospital Anxiety and Depression Scale (HADS) in different groups of Dutch subjects. Psychological Medicine.

[CR57] Stapersma, L., van den Brink, G., van der Ende, J., Szigethy, E. M., Beukers, R., Korpershoek, T. A., … Utens, E. (2018). Effectiveness of disease-specific cognitive behavioral therapy on anxiety, depression, and quality of life in youth with inflammatory bowel disease: A randomized controlled trial. *Journal of Pediatric Psychology, 43*, 967–980. 10.1093/jpepsy/jsy029. 10.1093/jpepsy/jsy029 PMC6147749 29850915

[CR58] Statistics Netherlands (2000). Standaarddefinitie allochtonen.

[CR59] Statistics Netherlands. (2010). Standaard Beroepen Classificatie 2010. The Hague: Statistics Netherlands. Retrieved from https://www.cbs.nl/nl-nl/onze-diensten/methoden/classificaties/onderwijs-en-beroepen/beroepenclassificatie–isco-en-sbc–/standaard-beroepenclassificatie-2010–sbc-2010–/downloaden-en-installeren-sbc-2010.

[CR60] Sweeney L, Moss-Morris R, Czuber-Dochan W, Meade L, Chumbley G, Norton C (2018). Systematic review: Psychosocial factors associated with pain in inflammatory bowel disease. Alimentary Pharmacology & Therapeutics.

[CR61] Szigethy, E., Bujoreanu, S. I., Youk, A. O., Weisz, J., Benhayon, D., Fairclough, D., … DeMaso, D. R. (2014). Randomized efficacy trial of two psychotherapies for depression in youth with inflammatory bowel disease. *Journal of the American Academy of Child and Adolescent Psychiatry, 53*, 726–735. 10.1016/j.jaac.2014.04.014 10.1016/j.jaac.2014.04.014 PMC4104185 24954822

[CR62] Szigethy, E., Kenney, E., Carpenter, J., Hardy, D. M., Fairclough, D., Bousvaros, A., … DeMaso, D. R. (2007). Cognitive-behavioral therapy for adolescents with inflammatory bowel disease and subsyndromal depression. *Journal of the American Academy of Child and Adolescent Psychiatry, 46*, 1290–1298. 10.1097/chi.0b013e3180f6341fs. 10.1097/chi.0b013e3180f6341f 17885570

[CR63] Szigethy, E., McLafferty, L., & Goyal, A. (2011). Inflammatory bowel disease. *Pediatric Clinics of North America, 58*, 903–920, x–xi. 10.1016/j.pcl.2011.06.007. 10.1016/j.pcl.2011.06.007 21855713

[CR64] Szigethy, E., Youk, A. O., Gonzalez-Heydrich, J., Bujoreanu, S. I., Weisz, J., Fairclough, D., … DeMaso, D. R. (2015). Effect of 2 psychotherapies on depression and disease activity in pediatric Crohn’s disease. *Inflammatory Bowel Diseases, 21*, 1321–1328. 10.1097/mib.0000000000000358. 10.1097/MIB.0000000000000358 PMC4437807 25822010

[CR65] Thompson, R. D., Craig, A., Crawford, E. A., Fairclough, D., Gonzalez-Heydrich, J., Bousvaros, A., … Szigethy, E. (2012). Longitudinal results of cognitive behavioral treatment for youths with inflammatory bowel disease and depressive symptoms. *Journal of Clinical Psychology in Medical Settings, 19*, 329–337. 10.1007/s10880-012-9301-8. 10.1007/s10880-012-9301-8 22699797

[CR66] Timbremont B, Braet C, Roelofs J (2008). Handleiding Children’s Depression Inventory (herziene versie).

[CR67] Turner, D., Otley, A. R., Mack, D., Hyams, J., de Bruijne, J., Uusoue, K., … Griffiths, A. M. (2007). Development, validation, and evaluation of a pediatric ulcerative colitis activity index: A prospective multicenter study. *Gastroenterology, 133*, 423–432. 10.1053/j.gastro.2007.05.029 17681163

[CR68] Utens EMWJ, van Rijen EHM, Erdman RAM, Verhulst FC (2000). Rotterdam’s Kwaliteit van Leven Interview.

[CR69] Van den Brink G, Stapersma L, El Marroun H, Henrichs J, Szigethy EM, Utens EM, Escher JC (2016). Effectiveness of disease-specific cognitive-behavioural therapy on depression, anxiety, quality of life and the clinical course of disease in adolescents with inflammatory bowel disease: Study protocol of a multicentre randomised controlled trial (HAPPY-IBD). BMJ Open Gastroenterology.

[CR70] Van der Does AJW (2002). BDI-II-NL Handleiding. De Nederlandse versie van de Beck Depression Inventory.

[CR71] Van der Zaag-Loonen HJ, Grootenhuis MA, Last BF, Derkx HHF (2004). Coping strategies and quality of life of adolescents with inflammatory bowel disease. Quality of Life Research.

[CR72] Van Tilburg, M. A., Claar, R. L., Romano, J. M., Langer, S. L., Drossman, D. A., Whitehead, W. E., … Levy, R. L. (2017). Psychological factors may play an important role in pediatric Crohn’s disease symptoms and disability. *Journal of Pediatrics, 184*, 94–100.e101. https://doi.org/10.1016/j.jpeds.2017.01.058 10.1016/j.jpeds.2017.01.058 PMC5407185 28238483

[CR74] Weisz JR, McCarty CA, Valeri SM (2006). Effects of psychotherapy for depression in children and adolescents: A meta-analysis. Psychological Bulletin.

[CR75] Zimmerman M, Martinez JH, Young D, Chelminski I, Dalrymple K (2013). Severity classification on the Hamilton Depression Rating Scale. Journal of Affective Disorders.

